# The effects of intracolonic EGF on mucosal growth and experimental carcinogenesis.

**DOI:** 10.1038/bjc.1991.53

**Published:** 1991-02

**Authors:** J. R. Reeves, R. C. Richards, T. Cooke

**Affiliations:** Department of Human Anatomy and Cell Biology, University of Liverpool, UK.

## Abstract

Although intra-luminal epidermal growth factor (EGF) may stimulate cell proliferation in the upper gastrointestinal tract, its role in the large bowel has not been established. We have therefore studied the effect of intra-rectal EGF administration on both normal growth and carcinogenesis in the rat colon. Colonic cancer was induced in rats with azoxymethane (10 mg kg-1 week-1 for 12 weeks s.c.) and controls dosed with saline. In each group, animals were randomised to receive EGF (12 nM, 0.8 nM or saline control) in 0.5 ml saline via a rectal tube daily for 24 weeks. At this time, crypt cell production rates (CCPRs) were determined at two sites in the colon: one of maximal and another of minimal exposure to EGF (5 cm and 10 cm from the anal margin respectively). No effects of EGF were seen at 10 cm. The lower dose of EGF gave CCPRs that mirrored the control values. The higher dose of EGF in the animals not treated with azoxymethane stimulated mucosal growth. Azoxymethane increased in CCPR, but this was suppressed by the high dose of EGF. These results suggest that (1) luminal EGF and azoxymethane independently increase the colonic CCPR and their combined effect is not synergistic but antagonistic; (2) EGF may have a role in normal epithelial growth, but does not potentiate colonic carcinogenesis in this model.


Br. J. Cancer (1991), 63, 223-226 © Macmillan Press Ltd., 1991

### The effects of intracolonic EGF on mucosal growth and experimental carcinogenesis

J.R. Reeves[1], R.C. Richards[1] & T. Cooke[2]

[1]*Department of Human Anatomy and Cell Biology, The University of Liverpool, PO Box 147, Liverpool L69 3BX;* [2]*Department of Surgery, University of Glasgow, Royal Infirmary, 10 Alexandra Parade, Glasgow G31 2ER, UK.*

**Summary** Although intra-luminal epidermal growth factor (EGF) may stimulate cell proliferation in the upper gastrointestinal tract, its role in the large bowel has not been established. We have therefore studied the effect of intra-rectal EGF administration on both normal growth and carcinogenesis in the rat colon. Colonic cancer was induced in rats with azoxymethane ($10\,mg\,kg^{-1}\,week^{-1}$ for 12 weeks s.c.) and controls dosed with saline. In each group, animals were randomised to receive EGF (12 nM, 0.8 nM or saline control) in 0.5 ml saline via a rectal tube daily for 24 weeks. At this time, crypt cell production rates (CCPRs) were determined at two sites in the colon: one of maximal and another of minimal exposure to EGF (5 cm and 10 cm from the anal margin respectively). No effects of EGF were seen at 10 cm. The lower dose of EGF gave CCPRs that mirrored the control values. The higher dose of EGF in the animals not treated with azoxymethane stimulated mucosal growth. Azoxymethane increased in CCPR, but this was suppressed by the high dose of EGF. These results suggest that (1) luminal EGF and azoxymethane independently increase the colonic CCPR and their combined effect is not synergistic but antagonistic; (2) EGF may have a role in normal epithelial growth, but does not potentiate colonic carcinogenesis in this model.

Epidermal Growth Factor (EGF) is a well characterised polypeptide that exhibits mitogenic effects on a wide range of cell types after binding to specific transmembrane receptors (Cohen, 1983). In the gastrointestinal tract EGF is secreted into the lumen by salivary (Starkey & Orth, 1977) and Brunner's glands (Elder *et al.*, 1978) and has been detected in the luminal contents and mucosa throughout the intestine (Schaudies *et al.*, 1989). Whilst the physiological role of EGF in the adult gut remains unclear, the demonstration of EGF receptors on intestinal epithelial cells (Forgue-Lafitte *et al.*, 1980) indicates that the peptide may be involved in intestinal homeostasis. *In vivo* studies that have examined the effects of EGF on the intestine can be categorised into those where the growth factor was administered systemically or those involving direct infusion into the gut lumen. Studies involving intravenous administration of large amounts of EGF on a short term basis have resulted in a stimulation of mucosal growth throughout the small and large intestine (Dembinski *et al.*, 1982; Goodlad *et al.*, 1987; Scheving *et al.*, 1980). However, as only small quantities of EGF are normally found in blood (Byyny *et al.*, 1974; Abe *et al.*, 1987) when compared to levels found in the gut lumen (Schaudies *et al.*, 1989) and as it is cleared from the circulation extremely quickly (Jorgensen *et al.*, 1988), luminal administration may be the more relevant approach for such studies. Work in this field has concentrated on the upper gastrointestinal tract using large quantities of EGF and has yielded conflicting results: some workers reported an EGF induced stimulation of mucosal growth (Dembinski *et al.*, 1982; Ulshen *et al.*, 1986), whilst others observed no significant mitogenic response (Goodlad *et al.*, 1987). Studies involving intracolonic or long term EGF administration have not been described.

As EGF can stimulate mucosal growth throughout the intestine and chemical carcinogenesis is promoted by hyperplasia of the target organ (Farber, 1981), especially in the colon (Williamson & Rainey, 1984), it has been suggested that EGF may play a role in intestinal carcinogenesis. Indeed, EGF may be particularly important in the development of colonic neoplasia as EGF receptors are over expressed in many large bowel carcinomas (Bradley *et al.*, 1986).

The work to be described here, therefore, investigates the effect of daily intracolonic EGF administration on the rat large bowel during experimental colorectal carcinogenesis and in untreated animals. Colonic epithelial growth was assessed after 24 weeks of treatment.

Correspondence: J.R. Reeves.
Received 28 March 1990; and in revised form 15 August 1990.

### Materials and methods

### *Experimental design*

Colonic cancer was induced in rats by subcutaneous injection of azoxymethane (10 mg/kg/week for 12 weeks). A control group was similarly dosed with isotonic saline. In each group, animals were randomised to receive one of three EGF doses: 0.8 nM, 12 nM or a saline control dissolved in 0.5 ml of saline (equal to 5, 75 or $0\,ng\,ml^{-1}$). This was administered via a 7.0 cm, 18 gauge stainless steel animal feeding tube (Popper and Sons Inc, Newhyde Park, NY 11040, USA) fully inserted through the anus into the colon. The treatment was on a 5 day per week basis for 24 weeks and commenced with the first azoxymethane injection.

### *Animals*

Forty-eight adult male Wistar rats weighing between 350 and 400 g, at the start of the experiment, were housed in groups of four with a 12 hour day/night cycle. Standard pelleted diet (Labsure CRM, Poole, Dorset, UK) and water were provided *ad libitum*.

### *EGF*

EGF was purified from mouse submaxillary glands (Savage & Cohen, 1972) and quantified using an extinction coefficient of 30.9 ($E^{1\%}_{1\,cm}$ at 280 nm). $^{125}I$ labelled preparations of this material specifically bound to human syncytiotrophoblast microvillous membranes; a rich source of the EGF receptor (Richards *et al.*, 1983). Superimposable competitive binding curves were obtained using three different EGF samples as the unlabelled ligand. These were: (1) the mouse EGF extracted for this study, (2) mouse EGF (a generous gift from H. Gregory, ICI, Alderley Park, Macclesfield, UK) and (3) recombinant urogastrone/human EGF (Amersham International plc, Amersham, UK). In a mitogenesis assay the EGF preparation used in this study stimulated cell division in mouse 3T3 fibroblasts at a concentration of 1.7 nM.

*Crypt cell production rates (CCPRs)*

The CCPRs were determined by stathmokinetic techniques as described in detail elsewhere (Goodlad & Wright, 1982).

On the morning before being killed, animals were given 1 mg $kg^{-1}$ vincristine sulphate (Oncovin, Eli Lilly, Basingstoke, UK) by intraperitoneal injection and killed at intervals ranging from 30 to 156 min. The colon was removed immediately, opened longitudinally to expose the mucosa, rinsed in isotonic saline, fixed in Carnoy's fluid for 6 h and stored in 70% ethanol.

Small pieces of colonic mucosa (2–3 mm square), from sampling sites at 5 and 10 cm from the anus in each rat, were stained using the Fuelgen reaction and single crypts were removed by microdissection. For each sample the number of arrested metaphases in 40 crypts was determined. The mean number of metaphases per crypt was plotted against time (the interval between vincristine injection and tissue fixation). The slope of the line was fitted by the method of least squares and gave the rate of entry of cells into mitosis or the crypt cell production rate. Differences in slopes were assessed by a two-tailed Student's $t$ test.

*Distribution studies with radiolabelled EGF*

$^{125}$I-labelled mouse EGF was prepared by the chloramine T method (Hunter & Greenwood, 1962) to a specific activity of 150 $\mu$Ci $\mu g^{-1}$. 0.5 ml of 4 nM radiolabelled EGF in saline was administered into the colon of adult male rats by the method described above. At intervals to 3 h the animals were killed and 1 ml of blood was removed by cardiac puncture. The intestine, liver, kidneys, spleen, thyroid and bladder contents were removed. A gamma hand monitor was used to ensure that no high concentrations of radioactivity remained in the carcass. The radioactivity of contaminated fur, bedding, faeces and excised tissues and fluids was measured in a LKB gamma counter.

The macromolecular nature of the $^{125}$I, in tissues and fluids containing sufficiently high levels, was assessed by trichloroacetic acid precipitation. 2 cm lengths of colon, the thyroid gland and individual faecal pellets were homogenised in 3 ml of 0.05 M acetic acid, spun at 6,000 $g$(av) for 30 s and the supernatant collected. The pellet was washed in a further 3 ml of acetic acid and respun and the supernatants were combined. 0.1 ml of supernatant, urine or blood plasma was added to 1.3 ml of a cold aqueous solution of 10% trichloroacetic acid and 1% phosphotungstic acid in 1.8 ml Eppendorf tubes. 0.1 ml of 1% bovine serum albumin (carrier protein) was added to make the total volume to 1.5 ml. The mixture was left on ice for 60 min, spun at 6,000 $g$(av) for 60 s and the radioactivity in the precipitate (bound to macromolecules) and supernatant (free $^{125}$I) was measured.

### Results

*Animals*

Before the end of the 24 week treatment period two animals died from extensive metastases; both had been treated with azoxymethane and rectal saline. All the remaining animals were used for the CCPR determinations except for one where post mitotic figures were present after the injection of vincristine. A total of eight tumours were produced that were solely in the animals treated with azoxymethane, but this was not sufficient for statistical analysis.

*Crypt cell production rates*

*(1) Non carcinogen group* The results for this group are summarised in Figure 1 and are expressed in cells per crypt per hour ± the standard error. At the 5 cm sampling site the CCPR for the saline controls and those receiving the lower dose of EGF were similar (5.54 ± 1.51 and 4.27 ± 0.55). The standard error associated with the saline treated group is

**Figure 1** Crypt cell production rates: Control group.

**Figure 2** Crypt cell production rates: Azoxymethane treated group.

high and is heavily influenced by one point that lies outside the 99.9% confidence limit of the regression line of the others. On omission of this point the CCPR falls to 4.09 ± 0.94. There is a statistically significant elevation of the CCPR to 8.08 ± 0.75 in the group treated with the higher concentration of EGF when compared to the low dosed group ($P<$ 0.001). When the higher dosed group is compared to the saline treated animals there is no significant difference unless the outlier is omitted ($P<0.01$).

At the 10 cm sampling site, the values of the CCPR did not differ significantly between the three groups with cell turnover of 4.27 ± 0.97, 5.36 ± 1.06 and 4.22 ± 1.27 for the controls, low and high dose EGF groups respectively.

*(2) Carcinogen group* The results are summarised in Figure 2. At the 5 cm sampling site the CCPRs for the animals receiving intra-rectal saline or the lower dose of EGF were elevated to 10.19 ± 0.97 and 9.44 ± 1.57 respectively. However, the rectal administration of 12 nM EGF resulted in a significant suppression of the CCPR to 4.22 ± 1.27 compared to the carcinogen animals receiving rectal saline ($P<$ 0.005) or the lower dose of EGF ($P<0.05$).

The CCPRs were not significantly different in all three groups at the 10 cm sampling site with values of 6.97 ± 1.20, 5.46 ± 1.13 and 4.48 ± 0.62 for the controls, low and high dose EGF groups respectively.

*Studies with $^{125}$I-labelled EGF*

The distribution of radioactivity at time points after rectal administration was extremely variable. However, it is possible to make an estimation of the movement of the peptide over a period of time. The following points were noted:

(1) A variable amount of radioactivity was lost from the colon immediately after rectal administration.

(2) Thirty min after rectal administration of $^{125}$I-labelled EGF, radioactivity was detected in the distal 8 cm of the colon. At 60 min a similar pattern of distribution was observed. At 180 min the radioactivity had shifted to the most distal 4 cm of the colon and rectum.

(3) The $^{125}I$ in the colonic lumen remained greater than 90% trichloroacetic acid precipitable.

(4) Radioactivity was detected in the stomach and the small intestine but not in anaesthetised rats suggesting that EGF may have been taken up by coprophagy.

(5) $^{125}I$ was found in the liver and kidneys, but not in sufficient quantities to perform a trichloroacetic acid precipitation. $^{125}I$ in the blood and urine was not bound to any macromolecules. The thyroid gland showed high levels of macromolecular radioactivity, but this was probably due to the incorporation of iodine into stored thyroglobulin.

### Discussion

This is the first study to involve chronic EGF administration and EGF dosing via the intracolonic route. From the results, it is clear that mucosal growth in the colon is influenced by this treatment.

The radiolabelled EGF tracing studies showed that this method of administration, via a rectal tube, gave a variable dose. It is not possible to determine the absolute dose of EGF received by a particular region of mucosa, nevertheless, it is clear that the 10 cm sampling site was rarely in contact with the rectally administered solution and if any contact occurred it was for a short period of time. In contrast, the 5 cm site was in contact with the labelled peptide after every dosing, for periods of up to 3 h.

A recent report has been helpful in determining the quantity of EGF normally present in the rat colon. In a luminal flush from the colon of 3 to 4 month old Sprague-Dawley rats, the EGF content was 100 to 170 picograms per gram body weight (Schaudies *et al.*, 1989). As our rats were 350–400 g, we can estimate the total quantity of EGF in the colonic lumen to be approximately 35 to 68 nanograms. In our study, rectal administration of 0.5 ml of 0.8 nM EGF (2.5 ng) may not have altered the growth factor levels, but the higher dose of 12 nM (35.5 ng) was likely to have caused a significant increase in the EGF concentration in the lumen of the lower colon. This may explain why the higher dose of EGF affected mucosal growth whereas the lower dose mirrored the values of the controls. However, caution must be exercised here. Although the radiolabel remaining in the colon during the tracing studies was >90% trichloroacetic acid precipitable, we have not determined whether all of this radioactivity represented biologically active growth factor available to colonic EGF receptors. In addition, many factors may contribute to the cumulative growth factor concentration experienced by a particular region of mucosa after long term rectal dosing. These could include the possibilities that EGF binds to faecal matter, may not penetrate colonic mucus layers or feedback mechanisms may exist that regulate endogeneous growth factor levels.

As no significant effects of EGF were observed at the 10 cm sampling site, it is suggested that the growth factor may have been having a local effect on the mucosa in the lower colon and rectum rather than gaining access to the blood and having a systemic mode of action on the entire gut. However, there is no evidence to suggesting that the two sites studied should be equally responsive to EGF. A small quantity of free $^{125}I$ was detected in the blood during the tracing studies suggesting that the rectal dosing may have resulted in some growth factor entering the circulation. Previous observations on the effects of continual intravenous infusion of EGF on gut growth, although performed under different conditions to our experiments, failed to produce a stimulatory effect in the colon with 3 $\mu$g per rat per day (Goodlad *et al.*, 1987). This produced a stable increase in plasma EGF concentration of approximately 115 pg ml$^{-1}$. In all our animals studied, the quantity of radiolabel detected in the blood was not sufficient to suggest that plasma EGF concentrations had increased by a fraction of these amounts. The maximum levels of radioactivity detected in the blood corresponded to less than 5 pg ml$^{-1}$ of EGF and in addition was less than 5% TCA precipitable. We therefore feel that any small amounts of EGF gaining access to the blood stream were probably insignificant.

From this evidence we would like to suggest that EGF did not cross the gut/blood barrier in significant quantities and probably exerted its effect locally via functional EGF receptors on the colonocytes. We would also like to suggest that these receptors are on the apical membranes, as tight junctions between adjacent cells should prevent the diffusion of luminal EGF to basolateral membrane receptors. No conclusive studies have been reported that describe receptor characteristics or position on colonic cells. In the small intestine their location is a matter of controversy with some reports describing EGF receptors predominantly localised to the brushborder membranes (Thompson, 1988), while others describe the receptors only on the basolateral membranes (Scheving *et al.*, 1989).

In our study, the colonic CCPRs of the groups treated with rectally administered saline were similar to previously reported values in both the carcinogen treated and control animals (Cooke *et al.*, 1984). The stimulatory effect of the carcinogen was not seen at the 10 cm position in this study; indeed, azoxymethane is known to produce a range of responses throughout the colon (Cooke *et al.*, 1984). The higher dose of EGF caused a significant stimulation of mucosal growth at the 5 cm sampling site in the control animals when compared to the animals receiving the lower dose. The significance is marginal when the comparison is made against the saline treated group (Figure 1); however, the large standard error of this determination is heavily influenced by one of the eight readings that deviates very strongly from the regression line. On omission of this point, the saline treated and high dose EGF treated groups are significantly different. We feel that this is a relatively common problem and has been noted previously (Goodlad *et al.*, 1987), and the removal of such outliers is justifiable on the grounds that parasites or other localised bowel infections could account for such deviations. It is therefore clear that the higher dose of EGF produced a stimulation of mucosal cell growth that may be due to the mitogenic effect of this growth factor. The same treatment, in the rats dosed with azoxymethane, resulted in a suppression of mucosal cell turnover from the normally elevated levels (Figure 2). This result was original and most surprising as other conditions that produce mucosal hypertrophy have a synergistic effect with azoxymethane (Williamson & Rainey, 1984).

There are few other studies that examine the relationship between luminal EGF and colonic carcinogenesis. However, sialadenectomy of rodents, a technique known to reduce circulating EGF levels, has been used in conjunction with a colonic carcinogen that is similar to azoxymethane, namely 1,2 dimethlyhydrazine (Li *et al.*, 1982). This treatment results in the production of fewer tumours than in the sham operated controls, and it has been suggested that this is a direct result of reduced EGF levels and therefore contradicts our data. However, the interpretation of this observation is not straightforward. A similar study (Gut *et al.*, 1987) indicated that sialadenectomy induced a compensatory 3-fold increase in duodenal EGF content which might actually increase luminal EGF concentration in the colon.

As yet, we do not have an explanation for the differing growth responses due to EGF of the azoxymethane treated colonic mucosa when compared to the normal colonic mucosa and we can only speculate. Suppression of growth by EGF, which has been reported in squamous cell carcinomas *in vitro* (Barnes, 1982; Kamata *et al.*, 1986) and *in vivo* with Ehrlich ascites cells (Lombardero *et al.*, 1986), may be related to high cell surface EGF receptor density. Thus, overexpression of EGF receptors in the azoxymethane treated mucosa may be responsible for the suppression of mucosal growth by EGF. Alternatively, it is now known that transforming growth factor alpha (TGF$\alpha$), a peptide that binds to the EGF receptor and has similar, but not identical biological activity to EGF (Burgess, 1989), is present in the colonic mucosa of man in concentrations that exceed those of EGF (Cartlidge & Elder, 1989). In addition, elevated levels of TGFα, EGF and other high molecular weight EGF like peptides have been identified in the intestinal mucosa of rats treated with the colonic carcinogen 1,2 dimethylhydrazine (Phylchenkov *et al.*, 1989). Thus, elevated levels of TGFα and other EGF like peptides may be present in the azoxymethane treated colonic mucosa. Addition of the rectally administered EGF may result in down regulation of mucosal EGF receptors and subsequent reduction in mucosal growth rates.

Further studies are currently underway to characterise EGF receptors in the colonic mucosa and to assess the effect of EGF on the numbers of tumours formed by azoxymethane.

### References

ABE, Y., SAGAWA, T., SAKAI, K. & KIMURA, S. (1987). Enzyme-linked immunosorbent assay (ELISA) for human epidermal growth factor (hEGF). *Clin. Chim. Acta.*, **168**, 87.

BARNES, D.W. (1982). Epidermal growth factor inhibits growth of A431 human epidermoid carcinoma in serum free culture. *J. Cell Biol.*, **93**, 1.

BRADLEY, S.J., GARFINKLE, G., WALKER, E., SALEM, E., CHEN, L.B. & STEELE, G. (1986). Increased expression of the epidermal growth factor receptor on human colon carcinoma cells. *Arch. Surg.*, **121**, 1242.

BURGESS, A.W. (1989). Epidermal growth factor and transforming growth factor a. *Br. Med. Bull.*, **45**, 401.

BYYNY, R.L., ORTH, D.N., COHEN, S. & DOYNE, E.S. (1974). Epidermal growth factor: effects of androgens and adrenergic agents. *Endocrinology*, **95**, 776.

CARTLIDGE, S.A. & ELDER, J.B. (1989). Transforming growth factor a and epidermal growth factor levels in normal human gastrointestinal mucosa. *Br. J. Cancer*, **60**, 657.

COHEN, S. (1983). The epidermal growth factor (EGF). *Cancer*, **51**, 1787.

COOKE, T., KIRKHAM, N., STAINTHORP, D.H., INMAN, C., GOETING, N. & TAYLOR, I. (1984). Detection of early neoplastic changes in experimentally induced colorectal cancer using scanning electron microscopy and cell kinetic studies. *Gut*, **25**, 748.

DEMBINSKI, A., GREGORY, H., KONTUREK, S.J. & POLANSKI, M. (1982). Trophic action of epidermal growth factor on the pancreas and gastroduodenal mucosa in rats. *J. Physiol. (Lond.)*, **325**, 35.

ELDER, J.B., WILLIAMS, G., LACEY, E. & GREGORY, H. (1978). Cellular localisation of human urogastrone/epidermal growth factor. *Nature*, **271**, 466.

FARBER, E. (1981). Chemical carcinogenesis. *N. Engl. J. Med.*, **305**, 1379.

FORGUE-LAFITTE, M., LABURTHE, M., CHAMBLIER, M., MOODY, A.J. & ROSSELIN, G. (1980). Demonstration of specific receptors for EGF-Urogastrone in isolated rat intestinal epithelial cells. *Febs Lett.*, **114**, 243.

GOODLAD, R.A., WILSON, T.J.G., LENTON, W., GREGORY, H., MCCULLAGH, K.G. & WRIGHT, N.A. (1987). Intravenous but not intragastric urogastrone-EGF is trophic to the intestine of parenterally fed rats. *Gut*, **28**, 573.

GOODLAD, R.A. & WRIGHT, N.A. (1982). Quantitative studies on epithelial replacement in the gut. In *Techniques in the Life Sciences, P2*, Digestive Physiology, Titchen, D.A. (ed.) *P212*, 1. Elsevier Biomedical: Ireland.

GUT, I.T., IVASHCHENKO, Y.D., GARMANCHUK, L.V., OSIPOVA, L.A. & BYKOREZ, A.I. (1987). Influence of epidermal growth factor on 1,2-Dimenthylhydrazine induced carcinogenesis in the rat intestinal mucosa. *Eksp. Onkol.*, **9**, 17.

HUNTER, W.M. & GREENWOOD, F.C. (1962). Preparation of iodine-131 labelled human growth hormone of high specific activity. *Nature*, **194**, 495.

JORGENSEN, P.E., POULSON, S.S. & NEXO, E. (1988). Distribution of i.v. administered epidermal growth factor in the rat. *Regul. Pept.*, **23**, 161.

KAMATA, N., CHIDA, K., RIKIMARU, K., HORIKOSHI, M., ENOMOTO, S. & KUROKI, T. (1986). Growth-inhibitory effects of epidermal growth factor and overexpression of its receptor on human squamous cell carcinoma in culture. *Cancer Res.*, **46**, 1648.

LI, A.K.C., SCHATTENKERK, M.E, DE VRIES, J.E., ROSS, J.S. & MALT, R.A. (1982). Saliva as a modifier of dimethylhydrazine induced colorectal cancer. In *Colonic carcinogenesis*, Malt, R.A. & Williamson, R.C.N. (eds) p. 261. MTP Press: Lancaster.

LOMBARDERO, J., PEREZ, R. & LAGE, A. (1986). Epidermal growth factor inhibits thymidine incorporation in Ehrlich ascites cells *in vitro*. *Neoplasma*, **33**, 423.

PHYLCHENKOV, A.A., GARMANCHUK, L.V., IVASHCHENKO, Y.D. & BYKOREZ, A.I. (1989). Production of EGF and EGF like peptides in the gastrointestinal mucosa of rats during 1,2-dimethylhydrazine induced carcinogenesis and compensatory regeneration. *Eksp. Onkol.*, **11**, 16.

RICHARDS, R.C., BEARDMORE, J.M., BROWN, P.J., MOLLOY, C.M. & JOHNSON, P.M. (1983). Epidermal growth factor receptors on isolated human placental syncytiotrophoblast plasma membrane. *Placenta*, **4**, 133.

SAVAGE, C.R. & COHEN, S. (1972). Epidermal growth factor and a new derivative. *J. Biol. Chem.*, **247**, 7609.

SCHAUDIES, P.R., GRIMES, G., DAVIS, D., RAO, R.K. & KOLDOVSKY, O. (1989). EGF content in the gastrointestinal tract of rats: effects of age and fasting/feeding. *Am. J. Physiol.*, **256**, G856.

SCHEVING, L.A., YEH, Y.C., TSAI, T.H. & SCHEVING, L.E. (1980). Circadian phase-dependent stimulatory effects of epidermal growth factor on deoxyribonucleic acid synthesis in the duodenum, jejunum, ileum, caecum, colon and rectum of the adult male mouse. *Endocrinology*, **106**, 1498.

SCHEVING, L.A., SHIURBA, R.A., NGUYEN, T.D. & GRAY, G.M. (1989). Epidermal growth factor receptor of the intestinal enterocyte. *J. Biol. Chem.*, **264**, 1735.

STARKEY, R.H. & ORTH, D.N. (1977). Radioimmunoassay of human epidermal growth factor (Urogastrone). *J. Clin. Endocrinol. Metab.*, **45**, 1144.

THOMPSON, J.F. (1988). Specific receptors for epidermal growth factor in rat intestinal microvillous membranes. *Am. J. Physiol.*, **254** (*Gastrointest. Liver Physiol.* **17**), G429.

ULSHEN, M.H., LYN-COOK, L.E. & RAASCH, R.H. (1986). Effects of intraluminal epidermal growth factor on mucosal proliferation in the small intestine of adult rats. *Gastroenterology*, **91**, 1134.

WILLIAMSON, R.C.N. & RAINEY, J.B. (1984). The relationship between intestinal hyperplasia and carcinogenesis. *Scand. J. Gastroenterol.*, **19** (Suppl 104), 57.


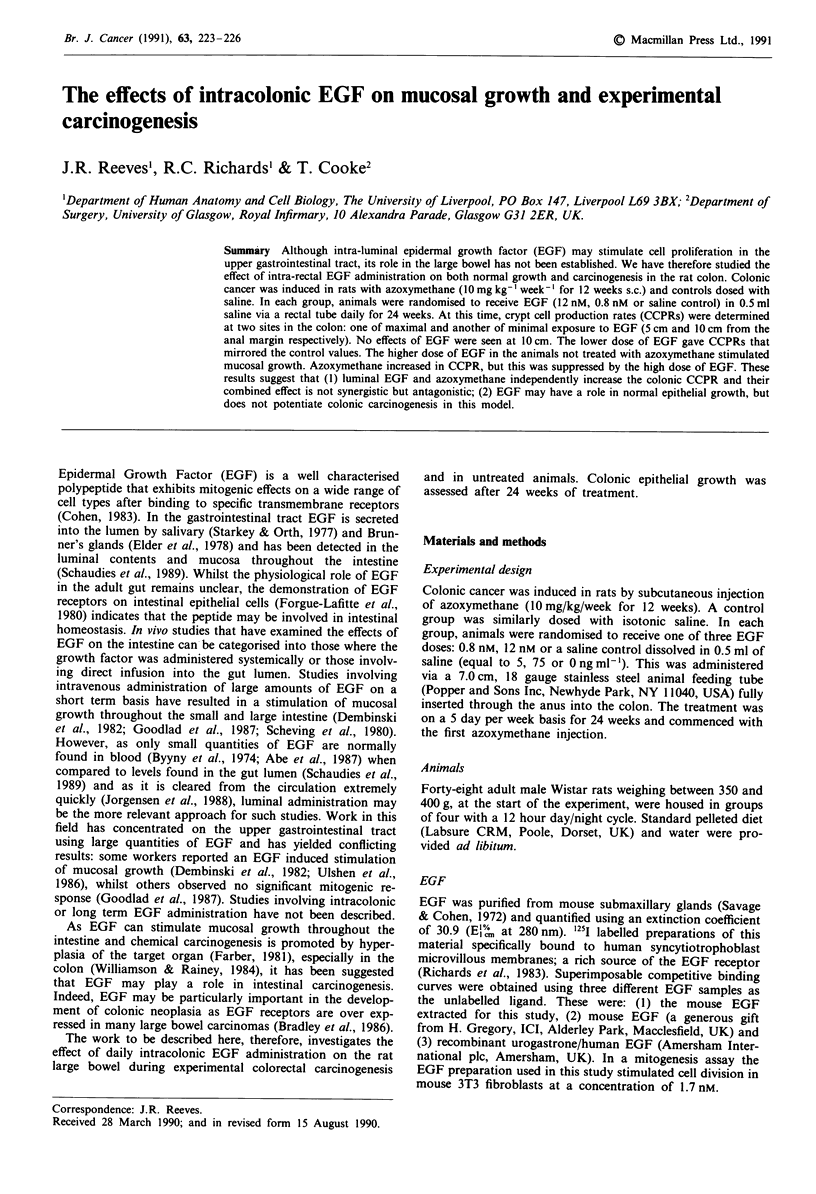

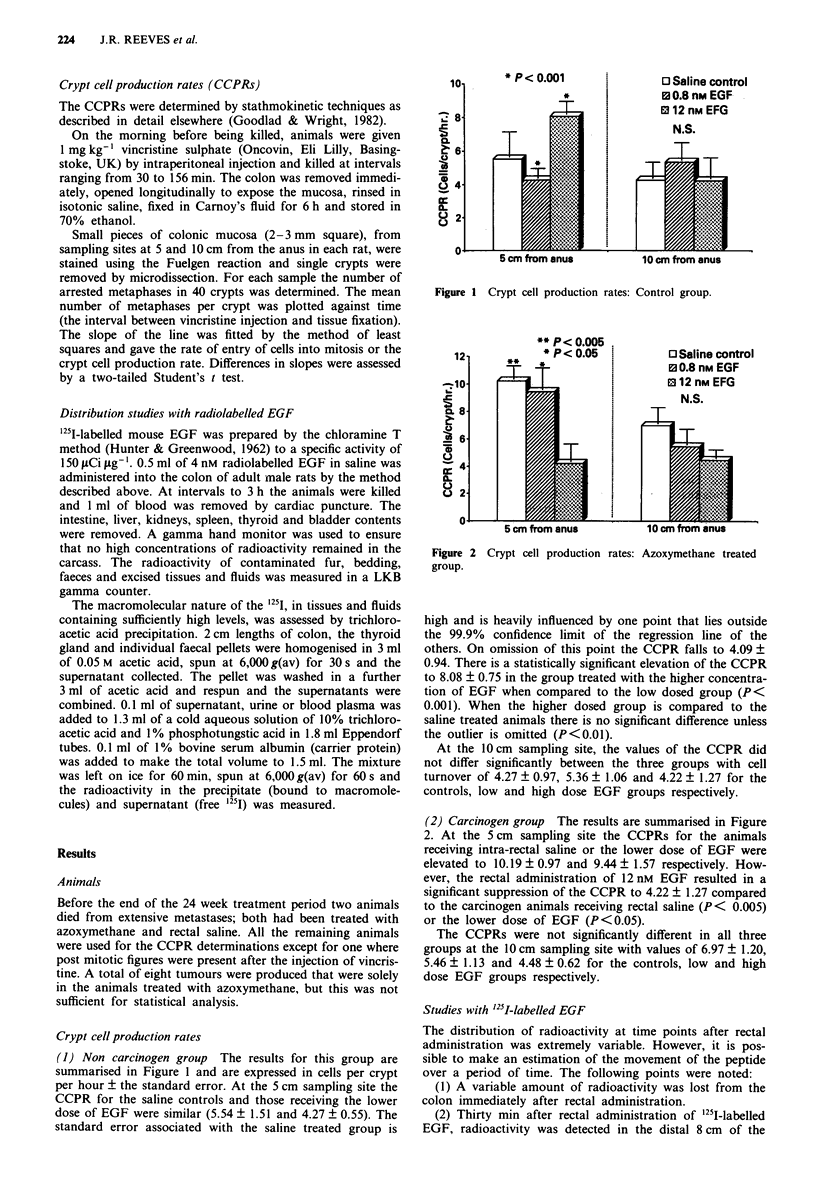

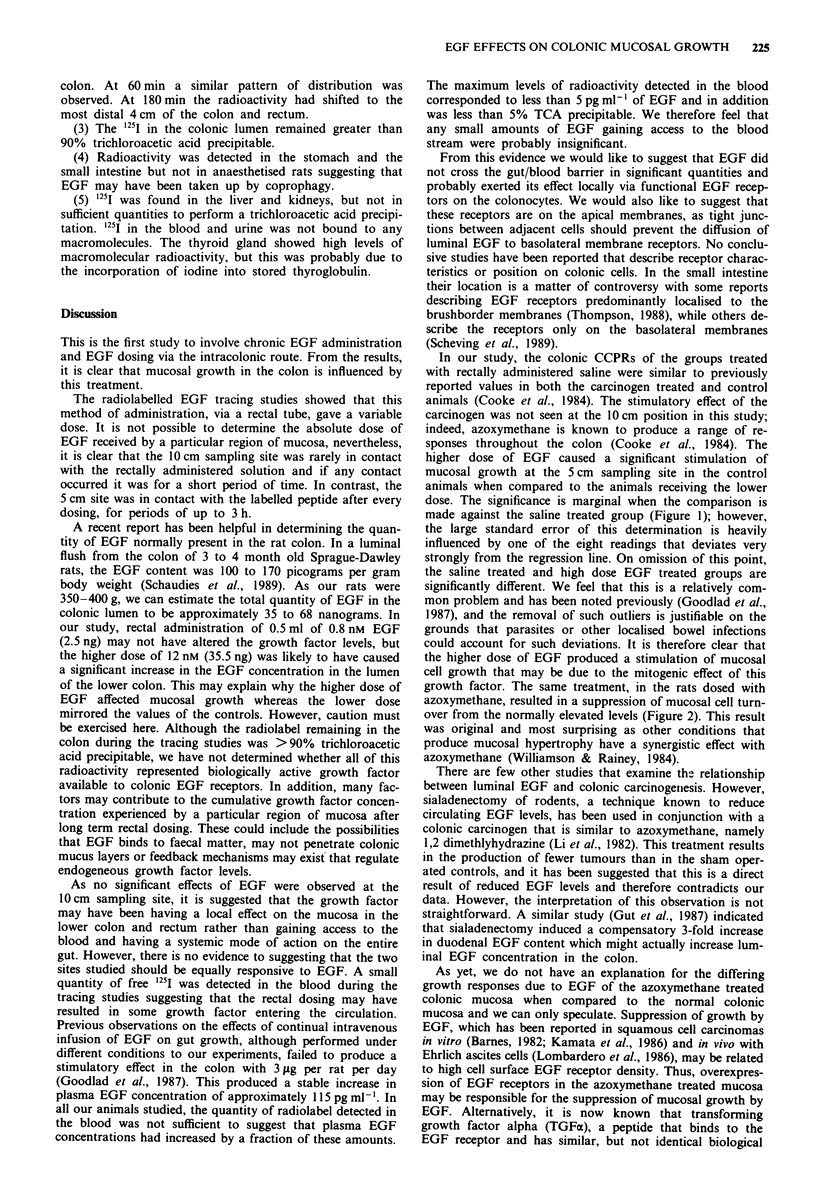

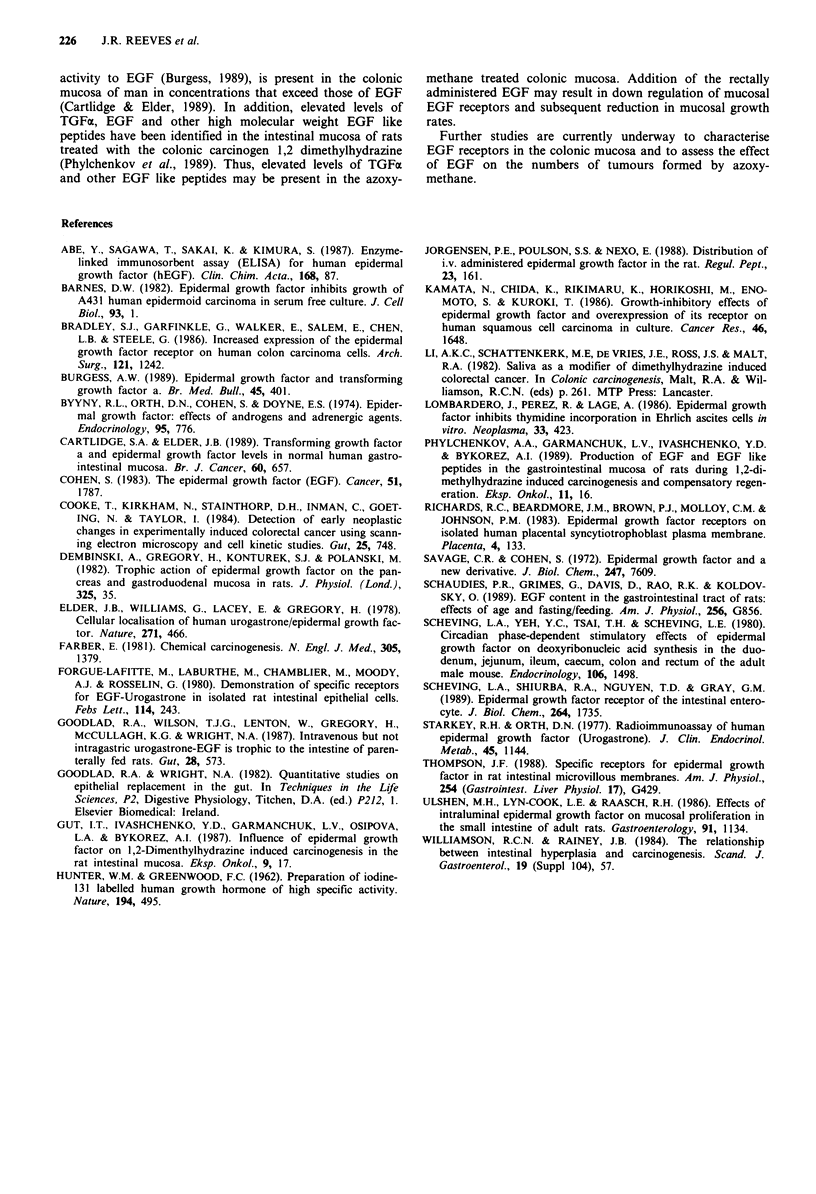


## References

[OCR_00495] Abe Y., Sagawa T., Sakai K., Kimura S. (1987). Enzyme-linked immunosorbent assay (ELISA) for human epidermal growth factor (hEGF).. Clin Chim Acta.

[OCR_00500] Barnes D. W. (1982). Epidermal growth factor inhibits growth of A431 human epidermoid carcinoma in serum-free cell culture.. J Cell Biol.

[OCR_00505] Bradley S. J., Garfinkle G., Walker E., Salem R., Chen L. B., Steele G. (1986). Increased expression of the epidermal growth factor receptor on human colon carcinoma cells.. Arch Surg.

[OCR_00511] Burgess A. W. (1989). Epidermal growth factor and transforming growth factor alpha.. Br Med Bull.

[OCR_00515] Byyny R. L., Orth D. N., Cohen S., Doyne E. S. (1974). Epidermal growth factor: effects of androgens and adrenergic agents.. Endocrinology.

[OCR_00520] Cartlidge S. A., Elder J. B. (1989). Transforming growth factor alpha and epidermal growth factor levels in normal human gastrointestinal mucosa.. Br J Cancer.

[OCR_00525] Cohen S. (1983). The epidermal growth factor (EGF).. Cancer.

[OCR_00531] Cooke T., Kirkham N., Stainthorp D. H., Inman C., Goeting N., Taylor I. (1984). Detection of early neoplastic changes in experimentally induced colorectal cancer using scanning electron microscopy and cell kinetic studies.. Gut.

[OCR_00535] Dembiński A., Gregory H., Konturek S. J., Polański M. (1982). Trophic action of epidermal growth factor on the pancreas and gastroduodenal mucosa in rats.. J Physiol.

[OCR_00541] Elder J. B., Williams G., Lacey E., Gregory H. (1978). Cellular localisation of human urogastrone/epidermal growth factor.. Nature.

[OCR_00546] Farber E. (1981). Chemical carcinogenesis.. N Engl J Med.

[OCR_00602] Fil'chenko A. A., Garmanchuk L. V., Ivashchenko Iu D., Bykorez A. I. (1989). Produktsiia épidermal'nogo faktora rosta i podobnykh emu polipeptidov slizistoi obolochke kishechnika krys pri indutsirovannom 1,2-dimetilgidrazinom kantserogeneze i kompensatornoi regeneratsii.. Eksp Onkol.

[OCR_00550] Forgue-Lafitte M. E., Laburthe M., Chamblier M. C., Moody A. J., Rosselin G. (1980). Demonstration of specific receptors for EGF--urogastrone in isolated rat intestinal epithelial cells.. FEBS Lett.

[OCR_00556] Goodlad R. A., Wilson T. J., Lenton W., Gregory H., McCullagh K. G., Wright N. A. (1987). Intravenous but not intragastric urogastrone-EGF is trophic to the intestine of parenterally fed rats.. Gut.

[OCR_00568] Gut I. T., Ivashchenko Iu D., Garmanchuk L. V., Osipova L. A., Bykorez A. I. (1987). Vliianie épidermal'nogo faktora rosta na kantserogenez, indutsirovannyi 1,2-dimetilgidrazinom v slizistoi obolochke kishechnika krys.. Eksp Onkol.

[OCR_00574] HUNTER W. M., GREENWOOD F. C. (1962). Preparation of iodine-131 labelled human growth hormone of high specific activity.. Nature.

[OCR_00579] Jørgensen P. E., Poulsen S. S., Nexø E. (1988). Distribution of i.v. administered epidermal growth factor in the rat.. Regul Pept.

[OCR_00586] Kamata N., Chida K., Rikimaru K., Horikoshi M., Enomoto S., Kuroki T. (1986). Growth-inhibitory effects of epidermal growth factor and overexpression of its receptors on human squamous cell carcinomas in culture.. Cancer Res.

[OCR_00597] Lombardero J., Pérez R., Lage A. (1986). Epidermal growth factor inhibits thymidine incorporation in Ehrlich ascites tumor cells in vivo.. Neoplasma.

[OCR_00609] Richards R. C., Beardmore J. M., Brown P. J., Molloy C. M., Johnson P. M. (1983). Epidermal growth factor receptors on isolated human placental syncytiotrophoblast plasma membrane.. Placenta.

[OCR_00615] Savage C. R., Cohen S. (1972). Epidermal growth factor and a new derivative. Rapid isolation procedures and biological and chemical characterization.. J Biol Chem.

[OCR_00619] Schaudies R. P., Grimes J., Davis D., Rao R. K., Koldovský O. (1989). EGF content in the gastrointestinal tract of rats: effect of age and fasting/feeding.. Am J Physiol.

[OCR_00631] Scheving L. A., Shiurba R. A., Nguyen T. D., Gray G. M. (1989). Epidermal growth factor receptor of the intestinal enterocyte. Localization to laterobasal but not brush border membrane.. J Biol Chem.

[OCR_00624] Scheving L. A., Yeh Y. C., Tsai T. H., Scheving L. E. (1980). Circadian phase-dependent stimulatory effects of epidermal growth factor on deoxyribonucleic acid synthesis in the duodenum, jejunum, ileum, caecum, colon, and rectum of the adult male mouse.. Endocrinology.

[OCR_00636] Starkey R. H., Orth D. N. (1977). Radioimmunoassay of human epidermal growth factor (urogastrone).. J Clin Endocrinol Metab.

[OCR_00641] Thompson J. F. (1988). Specific receptors for epidermal growth factor in rat intestinal microvillus membranes.. Am J Physiol.

[OCR_00646] Ulshen M. H., Lyn-Cook L. E., Raasch R. H. (1986). Effects of intraluminal epidermal growth factor on mucosal proliferation in the small intestine of adult rats.. Gastroenterology.

[OCR_00651] Williamson R. C., Rainey J. B. (1984). The relationship between intestinal hyperplasia and carcinogenesis.. Scand J Gastroenterol Suppl.

